# Interactive Effects of Polyamines and Plant Growth Regulators on Shoot Induction and Secondary Metabolism in *In Vitro* Shoot Cultures of *Echinacea* Species

**DOI:** 10.3390/molecules31040686

**Published:** 2026-02-17

**Authors:** Münüre Tanur Erkoyuncu

**Affiliations:** Department of Field Crops, Faculty of Agriculture, Selcuk University, Konya 42130, Türkiye; mtanur@selcuk.edu.tr

**Keywords:** caffeic acid derivatives, putrescine, spermidine, cytokinin–auxin interaction, species-specific responses, secondary metabolism

## Abstract

This study aimed to determine the regulatory role of polyamine–plant growth regulator (PGR) interactions on shoot development and caffeic acid derivatives (CADs) in *in vitro* shoot cultures of *Echinacea purpurea* and *Echinacea pallida*. Nodal explants were cultured under cytokinin-based (0.5 and 1.0 mg/L BAP) and auxin-containing (0.1 mg/L NAA) PGR combinations, supplemented with putrescine or spermidine at 50 and 100 mg/L. Shoot induction rate, number of shoots per explant, total phenolic content (TPC), total flavonoid content (TFC), total antioxidant capacity (TAC), and the contents of caffeic acid derivatives (caftaric acid, chlorogenic acid, caffeic acid, cichoric acid, and echinacoside) were quantitatively determined. The results revealed that shoot induction in both species was not statistically significant with respect to the PGR × polyamine interaction, and that shoot formation was primarily governed by the PGR composition. In contrast, pronounced species-specific differences were observed in secondary metabolism. In *E. purpurea*, overall phenolic and antioxidant indices remained relatively stable, whereas putrescine application, particularly under 0.5 mg/L BAP, induced a marked compositional redistribution of CADs, with maximum levels of cichoric acid (41.60 mg/g DW), chlorogenic acid (6.51 mg/g DW), and caffeic acid (0.23 mg/g DW). Conversely, *E. pallida* exhibited higher metabolic plasticity and responded more consistently to spermidine under auxin-containing media, where spermidine maximized chlorogenic acid (4.87 mg/g DW) and echinacoside (1.65 mg/g DW) accumulation alongside coordinated increases in TPC, TFC, and TAC. Overall, the results indicate that the polyamine-mediated modulation of caffeic acid derivatives in *Echinacea* species is strongly species- and hormone-dependent, underscoring the requirement for species-specific and PGR-conditioned optimization strategies in *in vitro* shoot culture systems.

## 1. Introduction

The *Echinacea* genus (particularly *E. purpurea*, *E. angustifolia*, and *E. pallida*) belongs to the Asteraceae family and comprises herbaceous, perennial species that are widely cultivated worldwide for both medicinal and ornamental purposes [1,2]. The roots, stems, and shoot tissues of *Echinacea* plants contain numerous bioactive compounds of therapeutic importance, including glycoproteins, alkamides, caffeic acid derivatives, polysaccharides, polyacetylenes, and essential oils [3,4]. These phytochemical constituents confer immunomodulatory, anti-inflammatory, antiviral, antifungal, and antioxidant properties to *Echinacea*, and several studies have also reported its anticancer and neuroprotective effects [5,6,7,8].

In recent years, increasing attention has been directed toward enhancing biomass production and bioactive compound yield in *Echinacea* species. In particular, the biosynthesis and accumulation of phenolic compounds, especially caffeic acid derivatives (CADs), has been shown to be highly sensitive to environmental conditions and the physiological status of the plant, a response that becomes especially pronounced under *in vitro* culture systems, where nutrient composition, growth regulators, and exogenously applied compounds precisely shape cellular metabolism and developmental processes [9,10,11]. In this context, polyamines—specifically putrescine, spermidine, and spermine—have garnered heightened interest in recent years due to their pivotal roles in plant stress responses, cellular defense mechanisms, and regeneration processes. Exogenous application of polyamines has been reported to improve antioxidant defense mechanisms, enhance stress resilience, and promote the accumulation of secondary metabolites in diverse plant species [12,13,14].

Polyamines are widely used in plant tissue culture systems to improve shoot regeneration, increase the number of shoots per explant, and enhance morphogenetic capacity. Numerous studies have demonstrated a strong relationship between endogenous putrescine levels and shoot development; conversely, inhibition of polyamine biosynthesis has been reported to restrict regeneration processes [15,16,17].

In addition to their roles in growth and development, polyamines also function as biologically derived chemical elicitors and biostimulants that enhance secondary metabolite production in plant tissue culture systems [12,13,18]. These compounds may stimulate cellular-defense-related responses, thereby indirectly affecting the synthesis and intracellular accumulation of pharmacologically valuable secondary metabolites. Polyamine applications have been shown to enhance the accumulation of secondary metabolites—particularly phenolic compounds and other secondary metabolite groups—in various *in vitro* culture systems through phenylpropanoid-metabolism-related processes and changes associated with antioxidant defense responses, rather than through direct mechanistic regulation [19,20,21]. However, these effects are highly dependent on polyamine type, concentration, treatment duration, and plant species, with lower concentrations generally promoting metabolite accumulation, whereas higher doses may suppress cell growth and secondary metabolism [22,23,24]. Such dose-dependent and species-specific responses highlight the importance of carefully optimized polyamine applications in medicinal plants with high phytochemical value, including *Echinacea* species.

Despite the growing body of literature on polyamine-mediated regulation of secondary metabolism, studies that comprehensively address the effects of polyamine applications on secondary metabolite profiles—particularly caffeic acid derivatives—in *Echinacea* species under *in vitro* shoot culture conditions and in interaction with plant growth regulators remain very limited.

Therefore, the aim of the present study was to investigate the effects of different concentrations of putrescine and spermidine on shoot development and secondary metabolite accumulation in *E. purpurea* and *E. pallida* under *in vitro* shoot culture conditions in interaction with plant growth regulators. In this context, the regulatory role of polyamine–plant growth regulator interactions were evaluated primarily with respect to shoot development and the accumulation of caffeic acid derivatives, and additionally in relation to complementary biochemical parameters, including total phenolic content (TPC), total flavonoid content (TFC), and total antioxidant capacity (TAC).

## 2. Results

### 2.1. Shoot Induction Responses to Plant Growth Regulator and Polyamine Treatments

#### 2.1.1. Shoot Induction in *E. purpurea*

**Figure 1:**
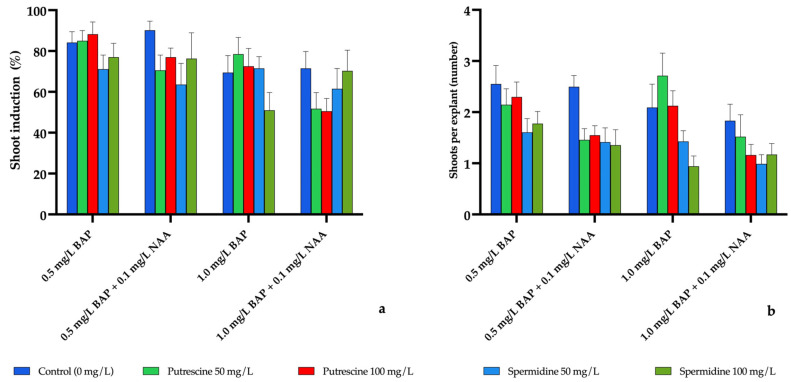
Effects of different plant growth regulator (PGR) combinations and polyamine applications on (**a**) shoot induction rate (%) and (**b**) number of shoots per explant in *E. purpurea*. Values are presented as mean ± standard error (SE). No statistically significant differences were detected among treatments (three-way ANOVA, *p* > 0.05).

In *E. purpurea*, shoot induction rate (%) and the number of shoots per explant exhibited a certain degree of variation depending on different plant growth regulator (PGR) combinations as well as polyamine type and dose (Figure 1). However, three-way ANOVA indicated that no statistically significant differences were detected among treatments for shoot induction rate or shoots per explant (*p* > 0.05). Therefore, treatment-related numerical variation is presented in Figure 1 and Supplementary Table S1 without further value-by-value description.

#### 2.1.2. Shoot Induction in *E. pallida*

**Figure 2:**
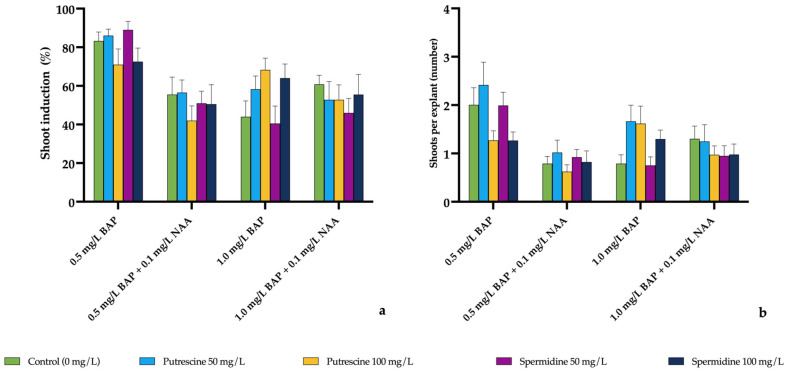
Effects of different plant growth regulator (PGR) combinations and polyamine applications on (**a**) shoot induction rate (%) and (**b**) number of shoots per explant in *E. pallida*. Values are presented as mean ± standard error (SE). No statistically significant differences were detected among treatments (three-way ANOVA, *p* > 0.05).

In *E. pallida*, shoot induction rate (%) and the number of shoots per explant exhibited numerical variation depending on different plant growth regulator (PGR) combinations as well as polyamine type and dose (Figure 2). However, three-way ANOVA indicated that no statistically significant differences were detected among treatments for shoot induction rate or shoots per explant (*p* > 0.05). Accordingly, the observed numerical variation is provided in Figure 2 and Supplementary Table S2 without detailed enumeration of individual values.

**Figure 3:**
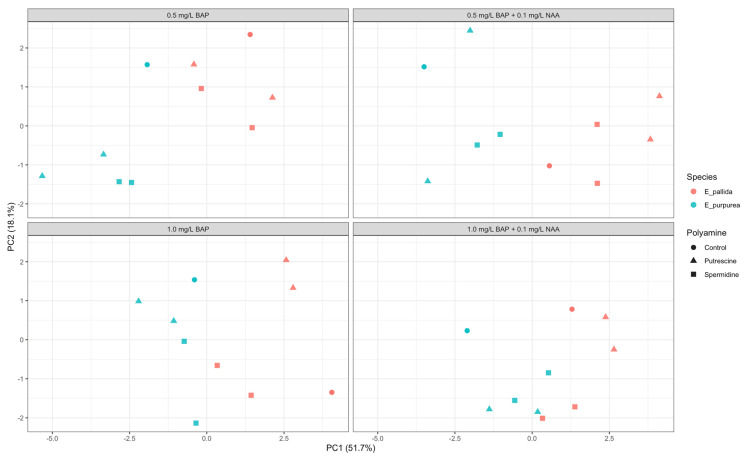
Principal component analysis (PCA) illustrating the multivariate distribution of shoot-induction-related morphological parameters in *E. purpurea* and *E. pallida* under different plant growth regulator (PGR) combinations and polyamine applications. PCA was performed by combining both species to visualize overall multivariate patterns, whereas univariate statistical analyses were conducted separately for each species.

To provide an exploratory overview of interspecific dispersion patterns in shoot-induction-related morphological parameters, principal component analysis (PCA) was performed using the combined dataset of *E. purpurea* and *E. pallida* (Figure 3). The analysis was based on shoot induction rate and number of shoots per explant, with all variables standardized prior to ordination. PC1 and PC2 explained 51.7% and 18.1% of the total variance, respectively. Across PGR backgrounds, samples of the two species tended to occupy different regions of the PCA space, reflecting species-associated multivariate positioning rather than strict separation. Variation along the PC2 axis primarily reflected within-species dispersion across treatments. Given that univariate analyses did not reveal statistically significant effects for shoot induction parameters, PCA results are presented here solely as a descriptive visualization of multivariate tendencies and not as inferential evidence of PGR × polyamine interactions.

### 2.2. Effects of Plant Growth Regulator and Polyamine Treatments on Biochemical Traits

#### 2.2.1. *E. purpurea*

##### Overview of Global Biochemical Indices

In *E. purpurea*, total phenolic content (TPC), total flavonoid content (TFC), and total antioxidant capacity (TAC) were evaluated in relation to different plant growth regulator (PGR) combinations as well as polyamine type and dose (Table 1). According to three-way ANOVA, none of these global indices showed statistically significant differences among treatments (*p* > 0.05); therefore, detailed numerical values are presented in Table 1 solely for reference and are not further interpreted.

##### Caffeic Acid Derivatives (CADs)

In *E. purpurea*, the contents of caffeic acid derivatives (caftaric acid, chlorogenic acid, caffeic acid, cichoric acid, and echinacoside) showed statistically significant variation when evaluated in relation to the combined effects of plant growth regulator (PGR) conditions, polyamine type, and dose (Table 2). According to the results of the three-way analysis of variance and Tukey’s multiple comparison test, all examined caffeic acid derivatives were significantly influenced by the PGR × polyamine type × polyamine dose interaction (* *p* < 0.05; ** *p* < 0.01).

Across treatments, cichoric acid represented the dominant caffeic acid derivative, exhibiting pronounced quantitative variation (20.77–41.60 mg/g DW). The highest cichoric acid accumulation was observed under 0.5 mg/L BAP in combination with 100 mg/L putrescine (41.60 ± 0.35 mg/g DW), whereas the lowest level occurred in the control treatment cultured on 1.0 mg/L BAP medium (20.77 ± 0.32 mg/g DW). Putrescine application under cytokinin-dominant conditions (0.5 mg/L BAP) was consistently associated with elevated levels of chlorogenic acid (up to 6.51 ± 0.04 mg/g DW) and caffeic acid (up to 0.23 ± 0.01 mg/g DW), indicating a treatment-specific enhancement of selected CADs. Caftaric acid content varied within a narrower range (1.81–3.75 mg/g DW), with maximum accumulation detected in the control treatment under 0.5 mg/L BAP + 0.1 mg/L NAA, whereas echinacoside levels displayed a more moderate but treatment-dependent response, reaching the highest value in the 50 mg/L putrescine treatment on 0.5 mg/L BAP medium (1.26 ± 0.02 mg/g DW).

#### 2.2.2. *E. pallida*

##### Overview of Global Biochemical Indices

In *E. pallida*, total phenolic content (TPC), total flavonoid content (TFC), and total antioxidant capacity (TAC) showed statistically significant differences when evaluated in relation to the combined effects of plant growth regulator (PGR) combinations, polyamine type, and dose (Table 3). According to the results of the three-way analysis of variance and Tukey’s multiple comparison test, the examined biochemical parameters were significantly influenced by the PGR × polyamine type × polyamine dose interaction (* *p* < 0.05; ** *p* < 0.01). Despite this statistical significance, these spectrophotometric indices are presented here as global descriptors of biochemical status rather than as primary indicators of treatment efficacy. Overall, higher TPC and TFC values were predominantly associated with control treatments and low-dose spermidine applications, particularly under NAA-containing media. For example, the highest TPC value was recorded in the control treatment cultured on 0.5 mg/L BAP + 0.1 mg/L NAA (44.35 ± 4.80 mg GAE/g DW), whereas the lowest value occurred in the control treatment under 1.0 mg/L BAP conditions (13.46 ± 0.31 mg GAE/g DW). Consistently, TFC values were higher under NAA-containing media, while control cultures on 1.0 mg/L BAP alone showed the lowest TFC (2.13 ± 0.06 mg QE/g DW). In contrast, TAC values exhibited a comparatively narrower range of variation across treatments, with the highest antioxidant capacity detected in the 100 mg/L spermidine treatment under 0.5 mg/L BAP + 0.1 mg/L NAA conditions (91.40 ± 0.11%). Accordingly, although TAC responses reached statistical significance, their discriminative power across treatments remained limited relative to compound-resolved CAD profiles and was therefore interpreted cautiously.

##### Caffeic Acid Derivatives (CADs)

In *E. pallida* the contents of caffeic acid derivatives (caftaric acid, chlorogenic acid, caffeic acid, cichoric acid, and echinacoside) showed statistically significant differences when evaluated in relation to the combined effects of plant growth regulator (PGR) conditions, polyamine type, and dose (Table 4). According to the results of the three-way analysis of variance and Tukey’s multiple comparison test, all examined caffeic acid derivatives were significantly influenced by the PGR × polyamine type × polyamine dose interaction (* *p* < 0.05; ** *p* < 0.01). Across treatments, multivariate differentiation was driven primarily by cichoric acid, chlorogenic acid, and echinacoside, which displayed the most treatment-sensitive and biologically informative shifts. Marked treatment-dependent variation was observed for cichoric acid (6.20–22.67 mg/g DW), with the highest accumulation detected in control cultures under 0.5 mg/L BAP + 0.1 mg/L NAA and similarly high levels under BAP-alone media with spermidine, whereas minimal levels occurred in control treatments cultured on 1.0 mg/L BAP. Chlorogenic acid exhibited the widest amplitude of variation (0.78–4.87 mg/g DW), reaching its maximum in the 100 mg/L spermidine treatment cultured on 0.5 mg/L BAP + 0.1 mg/L NAA medium, while markedly reduced levels were associated with putrescine treatments under the same hormonal background. Echinacoside accumulation showed a highly context-dependent pattern, ranging from non-detectable levels in some putrescine treatments to a maximum of 1.65 ± 0.03 mg/g DW in the 100 mg/L spermidine treatment under 1.0 mg/L BAP + 0.1 mg/L NAA conditions. In contrast, caftaric acid and caffeic acid displayed comparatively narrower response ranges and are therefore reported here as supportive components of the overall CAD profile rather than principal markers of treatment effects.

### 2.3. Species-Specific Multivariate Distribution of Shoot Induction and Biochemical Traits in Response to PGR × Polyamine Interactions (Heat Map Analysis)

**Figure 4:**
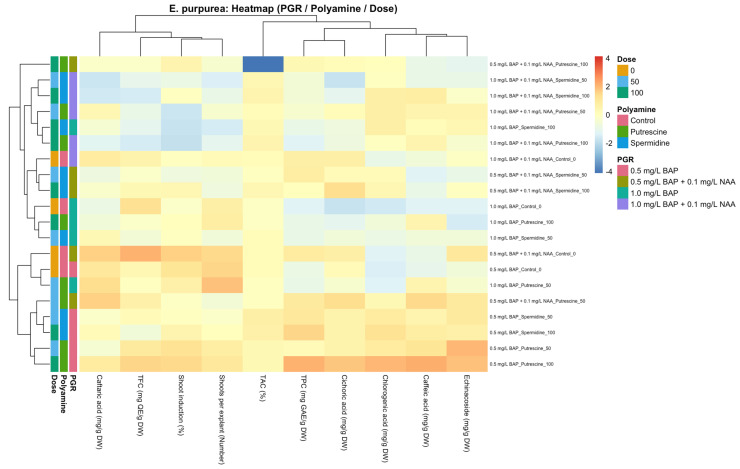
Heat map illustrating the combined effects of plant growth regulator (PGR) combinations, polyamine type, and dose on shoot-development-related morphological parameters (shoot induction rate and shoots per explant), total phenolic content (TPC), total flavonoid content (TFC), total antioxidant capacity (TAC), and individual caffeic acid derivatives (CADs) in *E. purpurea* under *in vitro* shoot culture conditions. Data were standardized using Z-score transformation prior to analysis. Hierarchical clustering was performed based on Euclidean distance using complete linkage. Side color bars indicate PGR combinations, polyamine types, and applied doses.

**Figure 5:**
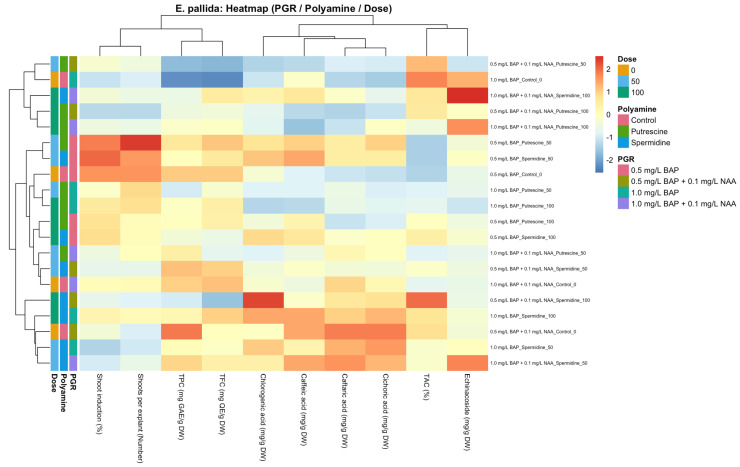
Heat map illustrating the combined effects of plant growth regulator (PGR) combinations, polyamine type, and dose on shoot-development-related morphological parameters (shoot induction rate and shoots per explant), total phenolic content (TPC), total flavonoid content (TFC), total antioxidant capacity (TAC), and individual caffeic acid derivatives (CADs) in *E. pallida* under *in vitro* shoot culture conditions. Data were standardized using Z-score transformation prior to analysis. Hierarchical clustering was performed based on Euclidean distance using complete linkage. Side color bars indicate PGR combinations, polyamine types, and applied doses.

Morphological traits related to shoot development, global biochemical indices (TPC, TFC, and TAC), and individual caffeic acid derivatives were evaluated using heat map analysis to visualize species-specific multivariate response patterns to plant growth regulator (PGR) composition and polyamine treatments. To avoid visual bias arising from interspecific differences in absolute values and variance structure, heat map analyses were performed separately for *E. purpurea* and *E. pallida* (Figure 4 and Figure 5). Heat maps were used as exploratory tools to summarize multivariate co-variation and clustering tendencies, rather than as independent evidence of statistical significance.

#### 2.3.1. *E. purpurea*

The heat map analysis of *E. purpurea* suggested that morphological parameters related to shoot development and CADs exhibited distinct distribution patterns depending on PGR, polyamine type, and application dose (Figure 4). Hierarchical clustering analysis revealed that polyamine types and doses applied under similar PGR combinations were grouped together, indicating that PGR background constituted the primary structuring factor of the multivariate response rather than individual polyamine effects. Under the same PGR combination, dose-dependent variation was predominantly reflected in the intensity patterns of caffeic acid derivatives, whereas shoot-induction-related morphological traits showed comparatively more constrained and less discriminatory variation across treatments. Global biochemical indices (TPC, TFC, and TAC) contributed only marginally to the overall clustering structure and were therefore interpreted as supportive descriptors rather than primary drivers of multivariate differentiation. When PGR combinations were compared, samples subjected to polyamine applications under NAA-containing media exhibited more pronounced clustering tendencies, particularly with respect to caffeic acid derivative profiles, whereas treatments cultured on media containing only BAP displayed a more heterogeneous distribution. In the heat map space, this was reflected as tighter within-group clustering under NAA-containing PGR combinations, alongside clearer separation of CAD intensity patterns across polyamine doses. Overall, the heat map highlights coordinated yet context-dependent multivariate responses, in which variation in caffeic acid derivatives represents the dominant source of differentiation, while morphological traits and global phenolic indices display secondary and more constrained contributions under the tested *in vitro* conditions.

#### 2.3.2. *E. pallida*

The heat map analysis of *E. pallida* revealed that morphological parameters associated with shoot development and CADs exhibited different intensity and distribution patterns depending on PGR, polyamine type, and application dose (Figure 5). Hierarchical clustering analysis demonstrated that treatments formed distinct clusters primarily based on PGR composition and polyamine applications, indicating a strong context dependency of multivariate responses in this species. Heat map results showed that polyamine applications in *E. pallida*, particularly in a dose-dependent manner, were associated with pronounced differences in the intensity patterns of both shoot development and CAD profiles. The occurrence of different clustering patterns under the same PGR combination with varying polyamine doses highlights the contribution of polyamine dose to multivariate patterning in *E. pallida*. When PGR combinations were compared, samples subjected to polyamine applications under NAA-containing media exhibited more consistent clustering patterns, whereas treatments cultured on media containing only BAP showed a more heterogeneous distribution, as evidenced by closer grouping of treatments and more coherent CADs intensity gradients under auxin-containing backgrounds (Figure 5). Across traits, variation in CADs represented the main contributors to multivariate differentiation, whereas TPC, TFC, and TAC showed comparatively weaker pattern contributions. The occurrence of similar clustering patterns between morphological traits and caffeic acid derivatives in certain treatment groups suggests coordinated, yet highly plastic multivariate, responses under the combined influence of PGR composition and polyamine dose.

## 3. Discussion

In both *E. purpurea* and *E. pallida*, shoot induction parameters did not differ significantly among PGR combinations, polyamine types, or application doses (*p* > 0.05). These results further indicate that the investigated polyamine treatments and PGR combinations did not produce statistically significant or consistent effects on shoot induction responses under *in vitro* culture conditions.

Consistent with previous reports, the present results indicate that PGR composition represents the primary regulatory determinant of shoot induction in *Echinacea* species. BAP-based or BAP-centered micropropagation protocols have been shown to induce limited yet reproducible shoot proliferation, with multiplication capacity strongly dependent on species identity and specific hormonal combinations [25,26]. Moreover, shoot regeneration efficiency has been shown to differ markedly among *Echinacea* species, with *E. pallida* frequently exhibiting a more restricted organogenic response [25,27,28]. Taken together, these findings corroborate that cytokinin-based basal protocols constitute the main drivers of shoot formation, while polyamines should be regarded as complementary, context-dependent regulators rather than direct inducers of organogenesis.

Although polyamines participate in key physiological processes, their effects *in vitro* are strongly dependent on species, tissue type, and applied concentration range [29,30]. While putrescine or spermidine treatments have been reported to enhance shoot number in certain plant species [15,29], numerous studies emphasize that polyamines more consistently influence shoot elongation, stress tolerance, or the reduction of hyperhydricity rather than shoot multiplication per se [16,17,31]. Additionally, advantageous polyamine effects are typically restricted to a limited optimal concentration range, while elevated doses may inhibit shoot regeneration in certain systems [32,33], underscoring the context-dependent and non-universal nature of polyamine-mediated responses.

In contrast to the relatively limited effects observed for morphological parameters, *E. pallida* exhibited significant variations in total phenolic content (TPC), total flavonoid content (TFC), and total antioxidant capacity (TAC) depending on polyamine type and application dose. This pattern suggests that biochemical traits in *E. pallida* respond more dynamically than morphogenetic parameters, indicating higher metabolic plasticity. The variable biochemical responses recorded in this species appear to be consistent with hormesis-like, dose-dependent response patterns, which have been frequently reported in plant systems [34,35]. Importantly, the present findings do not support a generalized enhancing effect of any single polyamine type or dose across all biochemical parameters. Rather, the observation that control treatments and lower spermidine doses produced relatively favorable TPC and TFC values under specific PGR combinations underscores the importance of interpreting polyamine effects within the context of the prevailing hormonal background [19,21,36]. Similarly, the pronounced dependence of TAC responses on PGR composition supports the notion that polyamines act not as primary elicitors but rather as context-dependent physiological regulators, with comparatively higher responses observed particularly under NAA-containing conditions [37,38]. These observations are consistent with studies showing that polyamines modulate ROS homeostasis in a concentration-dependent manner, promoting protective effects at optimal levels but inducing oxidative stress at higher doses [39,40,41]. Accordingly, the enhancement of biochemical parameters at specific polyamine doses under suitable PGR conditions, followed by attenuation at higher concentrations, indicates that polyamines operate within defined physiological limits as signaling and metabolic modulators in *E. pallida*.

Notably, in *E. purpurea*, the absence of statistically significant treatment effects on global biochemical indices (TPC, TFC, and TAC), despite pronounced and significant alterations in caffeic acid derivatives (CADs), suggests that polyamine treatments preferentially influenced phenolic composition rather than overall phenolic pool size. Under these conditions, antioxidant and phenolic capacities remained relatively stable, whereas variation was primarily reflected at the level of individual CADs. This divergence highlights the greater sensitivity of targeted HPLC-based analyses compared with bulk spectrophotometric assays for resolving treatment-dependent metabolic shifts.

Distinct regulatory strategies between species were particularly evident with respect to caffeic acid derivative (CAD) accumulation. In *E. purpurea*, CAD profiles showed a strong dependence on cytokinin level in combination with polyamine type and dose, indicating that the prevailing PGR background represents an important determinant of CAD modulation [42,43]. The increased accumulation of chlorogenic acid, caftaric acid, and cichoric acid under specific PGR combinations suggests that appropriate hormonal contexts may favor CAD biosynthesis. In this respect, the enhanced levels observed following putrescine application in cytokinin-containing media are consistent with previous reports describing compound-specific polyamine responses under defined hormonal conditions [44,45].

This interpretation is supported by the observation that 100 mg/L putrescine applied under 0.5 mg/L BAP conditions coincided with maximum chlorogenic acid, caffeic acid, and cichoric acid contents, suggesting a coordinated response of multiple CADs under a cytokinin-dominant background [13,46]. However, the concurrent decline in echinacoside content at higher putrescine concentrations is consistent with hormetic dose–response patterns reported for elicitor-like treatments, where stimulatory effects at moderate doses may shift toward inhibitory responses at elevated concentrations [47,48]. Similarly, the attainment of maximum caftaric acid levels in BAP + NAA control media, followed by reduced accumulation under spermidine application, indicates that certain CADs may be more sensitive to basal auxin–cytokinin balance than to polyamine treatment alone.

In contrast, CAD accumulation patterns in *E. pallida* were more heterogeneous and dose-responsive, indicating a higher degree of metabolic plasticity. Caftaric acid reached its highest levels under BAP + NAA control conditions, paralleling trends observed in *E. purpurea* and reinforcing the importance of basal auxin–cytokinin balance in regulating this compound [49]. Under specific PGR conditions, moderate increases in caftaric acid following low-dose spermidine application further suggest that spermidine may exert a stimulatory effect within a narrow concentration range. Conversely, reduced caftaric acid levels under high-dose putrescine treatments align with dose-dependent inhibitory effects commonly described in the hormesis literature [19,20,24].

Compound-specific responses further distinguish *E. pallida* from *E. purpurea*. In particular, the attainment of the highest chlorogenic and caffeic acid contents following 100 mg/L spermidine application under NAA-containing media, together with consistent increases in caffeic acid across different PGR backgrounds, suggests that spermidine may act as one of the dominant regulators of phenylpropanoid metabolism in *E. pallida* [20]. In contrast, cichoric acid accumulation was primarily associated with PGR composition and declined under higher cytokinin levels, consistent with previous reports describing pronounced medium- and tissue-specific variation for this compound [34,50]. Echinacoside, a characteristic CAD of *E. pallida*, exhibited a clearly polyamine-specific and dose-dependent response. The pronounced increase in echinacoside content under high-dose spermidine application, coupled with its absence in certain putrescine treatments, highlights functional differentiation between polyamine types. This response pattern agrees with earlier studies reporting that echinacoside accumulation is highly sensitive to *in vitro* culture conditions, particularly under auxin-containing regimes [51,52].

When both species are considered together, *E. purpurea* and *E. pallida* appear to differ in their regulatory strategies for CAD modulation. *E. purpurea* displays a more constrained and hormonally buffered CAD profile with selective sensitivity to putrescine, whereas *E. pallida* exhibits a more flexible and dose-responsive CAD pattern that is strongly influenced by spermidine type, concentration, and PGR background. These findings indicate that the effects of polyamines on phenolic metabolism emerge in a compound-specific and context-dependent manner.

Principal component analysis (PCA) and heat map approaches were employed in this study not for inferential statistical purposes, but as exploratory tools to describe the co-variation patterns and overall distribution trends of morphological and biochemical parameters across treatments. Such multivariate approaches are particularly useful in complex *in vitro* systems, allowing integrated interpretation of interacting morphological and biochemical traits beyond univariate responses. Similar PCA- and clustering-based approaches have been successfully applied to visualize coordinated morphological and metabolic responses in plant tissue culture systems and PGR-mediated experimental designs, even in cases where univariate morphological parameters showed limited statistical responsiveness [53]. Comparable applications have also been reported in other PGR-mediated *in vitro* systems [54,55]. In this context, the present findings emphasize that multivariate data exploration provides a complementary approach for the integrated interpretation of morphological and biochemical responses in *in vitro* culture systems, while not replacing univariate statistical inference.

## 4. Materials and Methods

### 4.1. Chemicals and Standards

All chemicals used throughout the study for plant tissue culture and biochemical analyses were of analytical grade and were obtained from Sigma-Aldrich (St. Louis, MO, USA), Merck (Darmstadt, Germany), and Duchefa. (Haarlem, The Netherlands). For the quantitative determination of caffeic acid derivatives, caffeic acid, caftaric acid, chlorogenic acid, cichoric acid, and echinacoside standards with a purity of ≥95% (HPLC grade) were purchased from Sigma-Aldrich (St. Louis, MO, USA). HPLC-grade acetonitrile and methanol were supplied by Merck (Darmstadt, Germany). Ultrapure water was produced using a Milli-Q PLUS 185 water purification system (Millipore, Milford, MA, USA). The polyamines putrescine and spermidine used in the experimental treatments were obtained from Duchefa Biochemie (Haarlem, The Netherlands).

### 4.2. Explant Preparation and In Vitro Culture Conditions

Seeds of *E. purpurea* and *E. pallida* were surface sterilized according to the protocol described by Erkoyuncu and Yorgancilar [56]. The sterilized seeds were aseptically sown on plant growth regulator (PGR)-free Murashige and Skoog [57] basal medium and maintained under controlled conditions for eight weeks. At the end of this period, healthy and contamination-free seedlings were selected, and nodal segments approximately 1.0–1.5 cm in length were excised and used as explant material.

Nodal explants were cultured on MS medium supplemented with BAP (0.5 or 1.0 mg/L), either alone or in combination with 0.1 mg/L NAA [25,58]. For each PGR treatment, the medium was further supplemented with putrescine or spermidine at concentrations of 50 and 100 mg/L, in addition to a polyamine-free control, with concentrations selected based on previously published *in vitro* studies [15,59]. Putrescine and spermidine solutions were sterilized by filtration through a 0.22 µm membrane filter and added to the culture media after autoclaving and cooling.

All media were solidified with 7 g/L agar and supplemented with 30 g/L sucrose as the carbon source. The experiments were conducted using three biological replicates, each consisting of three culture vessels containing 10 nodal explants per vessel. Cultures were incubated in a growth chamber under controlled conditions of 24 ± 2 °C temperature, 65% relative humidity, a 16/8 h light/dark photoperiod, and a light intensity of 50 µmol m^−2^ s^−1^, using a Sanyo MLR-351H growth cabinet. (Sanyo Electric Co., Osaka, Japan).

### 4.3. Shoot Induction and Growth Parameters

After four weeks of culture, shoot induction and development were evaluated using two primary parameters: shoot induction rate (%) and the number of shoots per explant.

The shoot induction rate (%) was calculated as the percentage of nodal explants producing multiple shoots relative to the total number of nodal explants initially cultured in each treatment. The number of shoots per explant was determined by dividing the total number of newly formed shoots by the number of nodal explants initially cultured.

### 4.4. Biochemical Analyses

For biochemical analyses, including total phenolic content (TPC), total flavonoid content (TFC), total antioxidant capacity (TAC), and caffeic acid derivatives (CADs), samples were prepared from *in vitro* grown shoots independently selected from each treatment. All biochemical analyses were performed using at least three independent replicates.

#### 4.4.1. Extraction Procedure

Plant material used for biochemical analyses was obtained from *in vitro* shoots harvested after four weeks of culture. The shoots were dried at 37 °C for 24 h and subsequently ground into a fine powder using a sterile porcelain mortar and pestle. Extraction was carried out using an ultrasound-assisted extraction (UAE) method. Briefly, 0.2 g of each dried and powdered sample was extracted with 70% HPLC-grade methanol by sonication in an ultrasonic bath for 15 min. The resulting extracts were filtered through a 0.45 µm membrane filter and stored at 4 °C until further analyses [56].

#### 4.4.2. Total Phenolic Content (TPC)

Total phenolic content was determined using the Folin–Ciocalteu method as described by Wu et al. [60]. For each sample, 100 µL of extract was mixed with 1 mL of diluted Folin–Ciocalteu reagent (1:4, *v*/*v*). After 5 min, 0.5 mL of 20% Na_2_CO_3_ was added. The mixtures were incubated at room temperature in the dark for 30 min, and absorbance was measured at 760 nm. TPC values were calculated using a gallic acid calibration curve (25–500 ppm, R^2^ ≈ 0.99) and expressed as mg gallic acid equivalents per g dry weight (mg GAE/g DW).

#### 4.4.3. Total Flavonoid Content (TFC)

Total flavonoid content was determined using the aluminum chloride colorimetric method according to Chang et al. [61]. The reaction mixture consisted of 500 µL extract, 1.5 mL methanol, 2.8 mL distilled water, 0.1 mL of 1 M potassium acetate, and 0.1 mL of 10% AlCl_3_. After incubation at room temperature for 30 min, absorbance was measured at 415 nm. TFC values were calculated using a quercetin calibration curve (25–1000 ppm, R^2^ ≈ 0.98) and expressed as mg quercetin equivalents per g dry weight (mg QE/g DW).

#### 4.4.4. Total Antioxidant Capacity (TAC)

Total antioxidant capacity was assessed using the DPPH radical scavenging assay as described by Erenler et al. [62]. For each sample, 300 µL of extract was mixed with 100 µL of 0.1 mM DPPH solution and incubated at room temperature in the dark for 30 min. Absorbance was measured at 517 nm, and DPPH radical scavenging activity was calculated using the following equation:

DPPH radical scavenging activity (%) = ((Acontrol − Asample)/Acontrol) × 100(1)

where Acontrol is the absorbance of the control reaction consisting of DPPH solution and methanol, and Asample is the absorbance of the reaction mixture containing DPPH and the sample extract. The decrease in absorbance reflects the DPPH radical scavenging activity of antioxidant compounds present in the extract.

#### 4.4.5. Quantitative Analysis of Caffeic Acid Derivatives (CADs)

**Figure 6:**
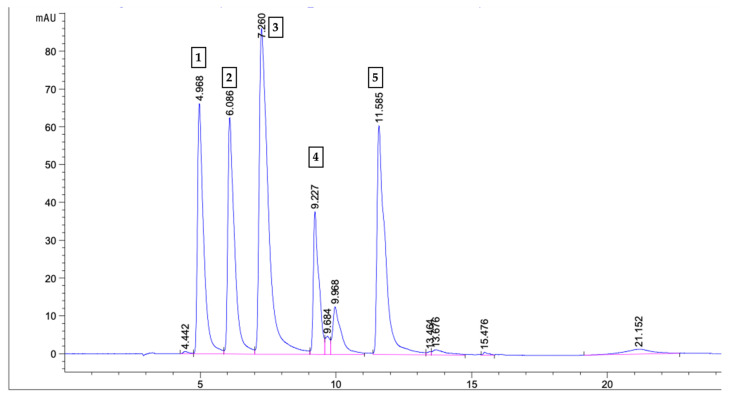
HPLC–DAD chromatogram (330 nm) of caffeic acid derivative standard compounds used for calibration. Standard compounds were injected individually and subsequently as mixed solutions at different concentrations for the construction of calibration curves. The numbered peaks correspond to (1) caftaric acid (Rt 4.99 min), (2) chlorogenic acid (Rt 6.09 min), (3) caffeic acid (Rt 7.26 min), (4) echinacoside (Rt 9.23 min), and (5) cichoric acid (Rt 11.58 min).

The quantitative analysis of caffeic acid derivatives, including caffeic acid, caftaric acid, chlorogenic acid, cichoric acid, and echinacoside, was carried out using an HPLC–DAD system following the protocol described by Erkoyuncu and Yorgancilar [56]. Standard solutions for each compound were prepared at concentrations of 1, 5, 10, 25, 50, and 100 ppm. HPLC–DAD chromatograms of each standard compound were recorded (Figure 6), and retention times were determined based on these chromatograms. Separate external calibration curves were constructed for each compound using peak areas. Linear regression analysis demonstrated a high degree of linearity for all caffeic acid derivatives (R^2^ ≥ 0.998). The concentrations of individual CADs in the samples were quantified using the corresponding calibration curves. The correlation coefficients (R^2^) for each compound are provided in Supplementary Table S3. Detailed information regarding the HPLC–DAD system, column type, mobile phase composition, and chromatographic conditions has been previously reported by Erkoyuncu and Yorgancilar [56].

### 4.5. Statistical Analysis

The experiment was conducted using a completely randomized design in a three-factor factorial arrangement. The experimental factors were plant growth regulator (PGR) treatment, polyamine type, and polyamine concentration. All data were analyzed by analysis of variance (ANOVA) using JMP Pro 18 software (SAS Institute Inc., Cary, NC, USA), taking interaction effects into account according to the experimental design. Considering the factorial structure of the experiment and the presence of control treatments at the 0 mg/L level, main effects and interactions were evaluated using an appropriate ANOVA model accounting for unequal cell sizes. When significant differences were detected, mean comparisons were performed using Tukey’s multiple comparison test. Results are presented as mean ± standard error (SE) based on at least three replicates.

Graphical representations were prepared using GraphPad Prism (version 10; GraphPad Software, San Diego, CA, USA). Multivariate analyses, including heatmap and principal component analysis (PCA), were performed in R (version 4.4.2; R Core Team, 2024). Prior to multivariate analyses, all biochemical variables were z-score standardized, centered around the mean, and scaled to unit variance to eliminate differences in measurement scales.

## 5. Conclusions

This study demonstrates that, under the tested *in vitro* conditions, polyamines do not function as primary inducers of shoot organogenesis in *E. purpurea* and *E. pallida*. Shoot induction was primarily governed by cytokinin-based PGR compositions, whereas polyamines acted as secondary, context-dependent modulators whose effects were constrained by species identity and hormonal background.

Although polyamine applications exerted limited effects on shoot-induction-related morphological traits, they substantially influenced secondary metabolism, particularly the composition of caffeic acid derivatives (CADs). The distinct metabolic responses observed between the two species indicate that polyamine-mediated regulation of phenylpropanoid metabolism is species- and context-dependent. Across both species, changes in CAD profiles emerged as the most sensitive indicators of PGR–polyamine interactions, largely independent of uniform shifts in total phenolic content. These findings suggest that polyamines modulate phenylpropanoid metabolism primarily through compound-level reprogramming rather than global phenolic accumulation.

Overall, the results indicate that modulation of secondary metabolite profiles in *Echinacea in vitro* cultures cannot be achieved through polyamine application alone. Instead, targeted enhancement of high value caffeic acid derivatives requires coordinated, species-specific optimization of polyamine type and dose within an appropriate PGR framework. This integrated approach provides a physiological basis for the rational design of *in vitro* culture systems aimed at improving the phytochemical quality of medicinal plants.

## Figures and Tables

**Table 1:** Effects of different plant growth regulator (PGR) combinations and polyamine applications on total phenolic content (TPC), total flavonoid content (TFC), and total antioxidant capacity (TAC) in *E. purpurea* ^1^.

PGR	Polyamine Type	Dose (mg/L)	TPC (mg GAE/g DW)	TFC (mg QE/g DW)	TAC (%)
0.5 mg/L BAP	Control	0	42.49 ± 0.17	8.53 ± 0.30	89.61 ± 0.08
	Putrescine	50	50.36 ± 0.32	9.54 ± 0.55	89.94 ± 0.12
		100	64.81 ± 5.41	10.52 ± 0.91	90.01 ± 0.05
	Spermidine	50	56.95 ± 3.10	8.19 ± 0.32	91.10 ± 0.08
		100	60.66 ± 7.72	6.94 ± 0.31	90.97 ± 0.07
0.5 mg/L BAP + 0.1 mg/L NAA	Control	0	55.42 ± 0.71	11.47 ± 0.95	89.46 ± 0.05
	Putrescine	50	56.04 ± 1.96	8.88 ± 0.26	89.12 ± 0.19
		100	51.12 ± 2.06	7.62 ± 0.08	78.31 ± 6.16
	Spermidine	50	54.97 ± 4.00	7.63 ± 0.37	89.06 ± 0.20
		100	48.88 ± 1.47	8.43 ± 0.51	90.26 ± 0.16
1.0 mg/L BAP	Control	0	39.07 ± 0.72	10.08 ± 0.74	88.94 ± 0.16
	Putrescine	50	41.51 ± 1.25	7.88 ± 0.60	89.16 ± 0.12
		100	41.41 ± 0.76	7.52 ± 0.84	88.88 ± 0.11
	Spermidine	50	41.85 ± 0.69	7.03 ± 0.27	89.46 ± 0.26
		100	41.77 ± 0.74	6.02 ± 0.01	89.98 ± 0.28
1.0 mg/L BAP + 0.1 mg/L NAA	Control	0	53.97 ± 0.17	8.75 ± 0.28	89.49 ± 0.03
	Putrescine	50	46.04 ± 1.09	6.30 ± 0.10	89.59 ± 0.09
		100	39.23 ± 0.33	5.38 ± 0.04	90.51 ± 0.17
	Spermidine	50	44.73 ± 0.05	6.18 ± 0.04	90.30 ± 0.07
		100	44.81 ± 0.34	5.38 ± 0.09	90.48 ± 0.10

^1^ Values are presented as mean ± standard error (SE). According to the results of the three-way analysis of variance, no statistically significant differences were detected among treatments for the evaluated parameters (*p* > 0.05).

**Table 2:** Effects of different plant growth regulator (PGR) combinations and polyamine applications on the contents of caffeic acid derivatives in *E. purpurea* ^1^.

PGR	Polyamine Type	Dose (mg/L)	Caftaric Acid ** (mg/g DW)	Chlorogenic Acid ** (mg/g DW)	Caffeic Acid ** (mg/g DW)	Cichoric Acid ** (mg/g DW)	Echinacoside ** (mg/g DW)
0.5 mg/L BAP	Control	0	3.39 ± 0.02 ^b^	2.41 ± 0.02 ^i^	0.14 ± 0.00 ^f^	32.07 ± 0.35 ^d^	0.71 ± 0.02 ^efg^
	Putrescine	50	2.54 ± 0.03 ^ef^	5.19 ± 0.05 ^c^	0.20 ± 0.01 ^ab^	34.30 ± 0.29 ^c^	1.26 ± 0.02 ^a^
		100	3.29 ± 0.03 ^c^	6.51 ± 0.04 ^a^	0.23 ± 0.01 ^a^	41.60 ± 0.35 ^a^	1.24 ± 0.02 ^a^
	Spermidine	50	2.66 ± 0.03 ^e^	5.17 ± 0.05 ^c^	0.18 ± 0.01 ^bcd^	34.00 ± 0.35 ^c^	1.03 ± 0.02 ^b^
		100	2.90 ± 0.03 ^d^	5.60 ± 0.05 ^b^	0.19 ± 0.01 ^bc^	34.00 ± 0.35 ^c^	0.97 ± 0.02 ^bc^
0.5 mg/L BAP + 0.1 mg/L NAA	Control	0	3.75 ± 0.03 ^a^	2.57 ± 0.02 ^i^	0.14 ± 0.01 ^ef^	35.30 ± 0.35 ^c^	1.05 ± 0.02 ^b^
	Putrescine	50	3.73 ± 0.03 ^a^	4.48 ± 0.04 ^e^	0.21 ± 0.01 ^ab^	38.80 ± 0.35 ^b^	1.03 ± 0.02 ^b^
		100	2.67 ± 0.03 ^e^	4.22 ± 0.03 ^f^	0.14 ± 0.01 ^ef^	31.80 ± 0.29 ^d^	0.60 ± 0.01 ^h^
	Spermidine	50	2.31 ± 0.02 ^g^	4.42 ± 0.04 ^e^	0.13 ± 0.01 ^f^	32.20 ± 0.35 ^d^	0.64 ± 0.01 ^gh^
		100	2.62 ± 0.03 ^e^	4.53 ± 0.05 ^e^	0.14 ± 0.01 ^ef^	38.97 ± 0.43 ^b^	0.83 ± 0.02 ^d^
1.0 mg/L BAP	Control	0	2.27 ± 0.03 ^g^	2.20 ± 0.03 ^j^	0.13 ± 0.01 ^f^	20.77 ± 0.32 ^h^	0.56 ± 0.01 ^hi^
	Putrescine	50	3.59 ± 0.03 ^b^	2.54 ± 0.03 ^i^	0.18 ± 0.01 ^bcd^	28.60 ± 0.29 ^e^	0.75 ± 0.01 ^def^
		100	2.40 ± 0.03 ^fg^	3.43 ± 0.03 ^g^	0.18 ± 0.01 ^bcd^	24.50 ± 0.23 ^g^	0.51 ± 0.01 ^i^
	Spermidine	50	2.97 ± 0.03 ^d^	3.13 ± 0.03 ^h^	0.15 ± 0.01 ^def^	27.30 ± 0.29 ^ef^	0.70 ± 0.01 ^fg^
		100	2.52 ± 0.03 ^ef^	5.00 ± 0.05 ^cd^	0.17 ± 0.01 ^cde^	27.10 ± 0.29 ^ef^	0.92 ± 0.02 ^c^
1.0 mg/L BAP + 0.1 mg/L NAA	Control	0	3.31 ± 0.03 ^c^	2.99 ± 0.03 ^h^	0.15 ± 0.01 ^def^	34.87 ± 0.38 ^c^	0.81 ± 0.02 ^d^
	Putrescine	50	3.00 ± 0.03 ^d^	5.01 ± 0.04 ^cd^	0.18 ± 0.01 ^bcd^	31.90 ± 0.29 ^d^	0.93 ± 0.02 ^c^
		100	2.09 ± 0.03 ^h^	4.08 ± 0.03 ^f^	0.18 ± 0.01 ^bcd^	26.50 ± 0.23 ^f^	0.76 ± 0.01 ^def^
	Spermidine	50	1.81 ± 0.02 ^i^	4.08 ± 0.03 ^f^	0.14 ± 0.01 ^ef^	20.80 ± 0.29 ^h^	0.63 ± 0.01 ^gh^
		100	1.85 ± 0.03 ^i^	4.96 ± 0.04 ^d^	0.19 ± 0.01 ^bc^	24.30 ± 0.23 ^g^	0.79 ± 0.02 ^de^

^1^ Values are presented as mean ± standard error (SE). Within the same column, means followed by different lowercase superscript letters indicate statistically significant differences according to the results of the three-way analysis of variance and Tukey’s multiple comparison test (** *p* < 0.01).

**Table 3:** Effects of different plant growth regulator (PGR) combinations and polyamine applications on total phenolic content (TPC), total flavonoid content (TFC), and total antioxidant capacity (TAC) in *E. pallida* ^1^.

PGR	Polyamine Type	Dose (mg/L)	TPC ** (mg GAE/g DW)	TFC ** (mg QE/g DW)	TAC ** (%)
0.5 mg/L BAP	Control	0	38.38 ± 0.26 ^ab^	5.79 ± 0.06 ^a^	88.89 ± 0.03 ^g^
	Putrescine	50	35.24 ± 0.21 ^ac^	5.84 ± 0.21 ^a^	88.82 ± 0.13 ^g^
		100	30.03 ± 0.13 ^bcd^	5.07 ± 0.21 ^ab^	89.88 ± 0.18 ^cdefg^
	Spermidine	50	30.41 ± 0.73 ^abcd^	5.28 ± 0.07 ^a^	88.82 ± 0.05 ^g^
		100	27.83 ± 0.95 ^bcd^	4.04 ± 0.14 ^abcd^	90.24 ± 0.02 ^bcdef^
0.5 mg/L BAP + 0.1 mg/L NAA	Control	0	44.35 ± 4.80 ^a^	4.66 ± 0.03 ^abc^	90.51 ± 0.17 ^abcd^
	Putrescine	50	17.62 ± 0.31 ^de^	2.70 ± 0.02 ^cd^	90.80 ± 0.13 ^abc^
		100	26.56 ± 0.23 ^bcde^	4.21 ± 0.13 ^abc^	90.34 ± 0.05 ^abcdef^
	Spermidine	50	38.97 ± 0.63 ^ab^	5.72 ± 0.20 ^a^	89.81 ± 0.13 ^cdefg^
		100	23.36 ± 0.26 ^cde^	2.88 ± 0.02 ^bcd^	91.40 ± 0.11 ^a^
1.0 mg/L BAP	Control	0	13.46 ± 0.31 ^e^	2.13 ± 0.06 ^d^	91.23 ± 0.12 ^ab^
	Putrescine	50	23.25 ± 0.38 ^cde^	4.38 ± 0.15 ^abc^	89.25 ± 0.05 ^cdefg^
		100	29.86 ± 0.64 ^bcd^	5.13 ± 0.26 ^ab^	89.38 ± 0.11 ^cdefg^
	Spermidine	50	31.01 ± 0.31 ^abcd^	4.57 ± 0.33 ^abc^	89.78 ± 0.06 ^cdefg^
		100	32.13 ± 0.42 ^abc^	4.57 ± 0.06 ^abc^	90.41 ± 0.17 ^abcde^
1.0 mg/L BAP + 0.1 mg/L NAA	Control	0	38.08 ± 0.72 ^ab^	5.90 ± 0.39 ^a^	89.38 ± 0.22 ^cdefg^
	Putrescine	50	33.66 ± 0.83 ^abc^	3.89 ± 0.06 ^abcd^	89.32 ± 0.11 ^cdefg^
		100	29.31 ± 0.93 ^bcd^	4.56 ± 0.10 ^abc^	89.58 ± 0.03 ^cdefg^
	Spermidine	50	37.59 ± 0.43 ^ab^	5.03 ± 0.05 ^ab^	89.78 ± 0.03 ^cdefg^
		100	26.18 ± 0.99 ^bcde^	5.24 ± 0.68 ^a^	90.37 ± 0.07 ^abcde^

^1^ Values are expressed as mean ± standard error. Different superscript lowercase letters indicate significant differences among treatments according to three-way ANOVA followed by Tukey’s test (** *p* < 0.01).

**Table 4:** Effects of different plant growth regulator (PGR) combinations and polyamine applications on the contents of caffeic acid derivatives in *E. pallida* ^1^.

PGR	Polyamine Type	Dose (mg/L)	Caftaric Acid ** (mg/g DW)	Chlorogenic Acid ** (mg/g DW)	Caffeic Acid ** (mg/g DW)	Cichoric Acid ** (mg/g DW)	Echinacoside ** (mg/g DW)
0.5 mg/L BAP	Control	0	1.48 ± 0.02 ^i^	1.97 ± 0.02 ^hi^	0.11 ± 0.01 ^def^	7.77 ± 0.15 ^k^	0.20 ± 0.01 ^ij^
	Putrescine	50	2.43 ± 0.03 ^d^	3.04 ± 0.03 ^e^	0.16 ± 0.01 ^ab^	18.40 ± 0.29 ^cd^	0.32 ± 0.01 ^efg^
		100	1.46 ± 0.02 ^i^	1.85 ± 0.03 ^hi^	0.14 ± 0.01 ^abcd^	9.00 ± 0.17 ^jk^	0.28 ± 0.01 ^fgh^
	Spermidine	50	2.43 ± 0.03 ^d^	3.49 ± 0.04 ^c^	0.17 ± 0.01 ^a^	15.80 ± 0.29 ^e^	0.45 ± 0.01 ^d^
		100	2.04 ± 0.03 ^f^	3.28 ± 0.03 ^d^	0.15 ± 0.01 ^abc^	13.30 ± 0.29 ^fg^	0.36 ± 0.01 ^e^
0.5 mg/L BAP + 0.1 mg/L NAA	Control	0	3.23 ± 0.03 ^a^	2.19 ± 0.03 ^g^	0.17 ± 0.01 ^a^	22.67 ± 0.38 ^a^	0.31 ± 0.01 ^efg^
	Putrescine	50	1.59 ± 0.02 ^i^	0.78 ± 0.02 ^m^	0.10 ± 0.01 ^ef^	8.80 ± 0.23 ^jk^	nd
		100	1.30 ± 0.02 ^k^	1.52 ± 0.02 ^j^	0.10 ± 0.01 ^ef^	7.90 ± 0.17 ^k^	0.45 ± 0.01 ^d^
	Spermidine	50	1.94 ± 0.02 ^fg^	1.82 ± 0.03 ^i^	0.13 ± 0.01 ^bcde^	11.60 ± 0.23 ^h^	0.25 ± 0.01 ^ghi^
		100	2.51 ± 0.03 ^c^	4.87 ± 0.05 ^a^	0.13 ± 0.01 ^bcde^	17.07 ± 0.32 ^de^	0.23 ± 0.01 ^hij^
1.0 mg/L BAP	Control	0	1.32 ± 0.02 ^jk^	1.08 ± 0.02 ^l^	0.13 ± 0.01 ^bcde^	6.20 ± 0.12 ^l^	1.07 ± 0.02 ^c^
	Putrescine	50	1.81 ± 0.02 ^gh^	1.31 ± 0.02 ^k^	0.11 ± 0.01 ^def^	9.10 ± 0.17 ^jk^	0.18 ± 0.01 ^j^
		100	1.77 ± 0.02 ^h^	0.79 ± 0.02 ^m^	0.10 ± 0.01 ^ef^	9.77 ± 0.20 ^ij^	nd
	Spermidine	50	2.93 ± 0.03 ^b^	3.45 ± 0.04 ^c^	0.14 ± 0.01 ^abcd^	21.30 ± 0.29 ^a^	0.49 ± 0.02 ^d^
		100	2.73 ± 0.03 ^c^	3.85 ± 0.04 ^b^	0.17 ± 0.01 ^a^	19.67 ± 0.32 ^bc^	0.35 ± 0.01 ^e^
1.0 mg/L BAP + 0.1 mg/L NAA	Control	0	2.72 ± 0.03 ^c^	2.00 ± 0.03 ^h^	0.12 ± 0.01 ^cdef^	14.40 ± 0.29 ^f^	0.20 ± 0.01 ^ij^
	Putrescine	50	2.27 ± 0.03 ^e^	1.33 ± 0.02 ^k^	0.12 ± 0.01 ^cdef^	13.30 ± 0.23 ^fg^	0.17 ± 0.01 ^j^
		100	1.47 ± 0.02 ^i^	1.45 ± 0.02 ^jk^	0.09 ± 0.01 ^f^	13.00 ± 0.23 ^g^	1.23 ± 0.02 ^b^
	Spermidine	50	3.11 ± 0.03 ^a^	2.69 ± 0.03 ^f^	0.17 ± 0.01 ^a^	19.80 ± 0.29 ^b^	1.29 ± 0.02 ^b^
		100	2.05 ± 0.03 ^f^	2.64 ± 0.03 ^f^	0.15 ± 0.01 ^abc^	10.30 ± 0.23 ^hi^	1.65 ± 0.03 ^a^

^1^ Values are expressed as mean ± standard error. Different superscript lowercase letters indicate significant differences among treatments according to three-way ANOVA followed by Tukey’s test (** *p* < 0.01). (nd: not detected).

## Data Availability

The original contributions presented in this study are included in the article/Supplementary Material. Further inquiries can be directed to the corresponding author.

## Supplementary Materials

The following supporting information can be downloaded at: https://www.mdpi.com/article/10.3390/molecules31040686/s1, Table S1: Effects of plant growth regulators (PGRs) and polyamine treatments on shoot organogenesis in *Echinacea purpurea*; Table S2: Effects of plant growth regulators (PGRs) and polyamine treatments on shoot organogenesis in *Echinacea pallida*; Table S3: Calibration parameters of caffeic acid derivatives used for HPLC–DAD quantification.

## Abbreviations

The following abbreviations are used in this manuscript:

BAP	6-Benzylaminopurine
CADs	Caffeic acid derivatives
DW	Dry weight
HPLC–DAD	High-performance liquid chromatography with diode-array detection
NAA	1-Naphthaleneacetic acid
PCA	Principal component analysis
PGR	Plant growth regulator
SE	Standard error
TAC	Total antioxidant capacity
TFC	Total flavonoid content
TPC	Total phenolic content
