# Supplementary material for: Interactive Effects of Polyamines and Plant Growth Regulators on Shoot Induction and Secondary Metabolism in In Vitro Shoot Cultures of Echinacea Species
Source: Molecules. 2026 Feb 17;31(4):686. doi: 10.3390/molecules31040686 (PMC12943704; doi:10.3390/molecules31040686)
Supplement: Supplementary file 1 [file molecules-31-00686-s001.zip › Supplementary Materials_S1_S2_S3.pdf]

Table S1. Effects of plant growth regulators (PGRs) and polyamine treatments on shoot organogenesis in *Echinacea purpurea*[1]

PGR	Polyamine Type	Dose (mg/L)	Shoot induction (%)	Shoots per explant (Number)
0.5 mg/L BAP	Control	0	84.18 ± 1.67	2.55 ± 0.11
	Putrescine	50	85.00 ± 1.59	2.14 ± 0.10
		100	88.25 ± 1.89	2.30 ± 0.09
	Spermidine	50	71.11 ± 2.18	1.60 ± 0.08
		100	77.00 ± 2.14	1.77 ± 0.08
0.5 mg/L BAP + 0.1 mg/L NAA	Control	0	90.14 ± 1.42	2.49 ± 0.07
	Putrescine	50	70.50 ± 2.38	1.46 ± 0.07
		100	77.00 ± 1.41	1.55 ± 0.06
	Spermidine	50	63.58 ± 3.27	1.41 ± 0.09
		100	76.25 ± 4.02	1.35 ± 0.10
1.0 mg/L BAP	Control	0	69.44 ± 2.62	2.09 ± 0.14
	Putrescine	50	78.50 ± 2.59	2.71 ± 0.14
		100	72.50 ± 2.77	2.12 ± 0.09
	Spermidine	50	71.50 ± 1.82	1.43 ± 0.07
		100	51.00 ± 2.75	0.94 ± 0.06
1.0 mg/L BAP + 0.1 mg/L NAA	Control	0	71.50 ± 2.62	1.83 ± 0.10
	Putrescine	50	51.75 ± 2.49	1.52 ± 0.14
		100	50.50 ± 1.98	1.16 ± 0.07
	Spermidine	50	61.50 ± 3.14	0.99 ± 0.06
		100	71.06 ± 3.38	1.21 ± 0.07

[1]Values are presented as mean ± standard error (SE). According to the results of the three-way analysis of variance, no statistically significant differences were detected among treatments for the evaluated parameters ($p > 0.05$).

Table S2. Effects of plant growth regulators (PGRs) and polyamine treatments on shoot organogenesis in *Echinacea pallida*[1]

PGR	Polyamine Type	Dose (mg/L)	Shoot induction (%)	Shoots per explant (Number)
0.5 mg/L BAP	Control	0	83.25 ± 1.47	2.01 ± 0.11
	Putrescine	50	86.00 ± 1.07	2.42 ± 0.15
		100	71.00 ± 2.57	1.27 ± 0.06
	Spermidine	50	89.00 ± 1.39	1.99 ± 0.09
		100	72.50 ± 2.23	1.27 ± 0.06
0.5 mg/L BAP + 0.1 mg/L NAA	Control	0	55.50 ± 2.86	0.79 ± 0.05
	Putrescine	50	56.50 ± 2.07	1.02 ± 0.08
		100	42.00 ± 2.41	0.62 ± 0.04
	Spermidine	50	51.00 ± 1.97	0.92 ± 0.05
		100	50.50 ± 3.19	0.82 ± 0.07
1.0 mg/L BAP	Control	0	44.00 ± 2.59	0.79 ± 0.06
	Putrescine	50	58.25 ± 2.16	1.67 ± 0.11
		100	68.25 ± 1.93	1.62 ± 0.11
	Spermidine	50	40.50 ± 2.86	0.75 ± 0.06
		100	64.00 ± 2.31	1.30 ± 0.06
1.0 mg/L BAP + 0.1 mg/L NAA	Control	0	60.75 ± 1.50	1.30 ± 0.08
	Putrescine	50	52.75 ± 3.02	1.25 ± 0.11
		100	52.75 ± 2.47	0.97 ± 0.06
	Spermidine	50	46.00 ± 2.37	0.95 ± 0.07
		100	55.50 ± 3.31	0.98 ± 0.07

[1]Values are presented as mean ± standard error (SE). According to the results of the three-way analysis of variance, no statistically significant differences were detected among treatments for the evaluated parameters ($p > 0.05$).

Table S3. Calibration parameters of caffeic acid derivatives used for HPLC–DAD quantification

Compound	Concentration range (ppm)	Regression equation	R^2
Caftaric acid	1–100	y = 10.339x − 2.6072	0.9998
Chlorogenic acid	1–100	y = 10.64x − 4.6209	0.9999
Caffeic acid	1–100	y = 19.931x − 20.947	0.9982
Echinacoside	1–100	y = 5.3007x − 2.4097	0.9982
Cichoric acid	1–100	y = 12.569x − 9.6378	0.9997
